# Long-term kidney function of patients discharged from hospital after an intensive care admission: observational cohort study

**DOI:** 10.1038/s41598-021-89454-3

**Published:** 2021-05-11

**Authors:** Ryan W. Haines, Jonah Powell-Tuck, Hugh Leonard, Siobhan Crichton, Marlies Ostermann

**Affiliations:** 1 William Harvey Research Institute, Queen Mary University of London, London, EC1M 6BQ UK; 2 Department of Critical Care, King’s College London, Guy’s and St Thomas’ NHS Foundation Trust, London, SE1 7EH UK; 3 Department of Renal Medicine, Guy’s and St Thomas’ NHS Foundation Trust, London, SE1 9RT UK; 4 MRC Clinical Trials Unit at University College London, London, UK

**Keywords:** Epidemiology, Outcomes research, Acute kidney injury

## Abstract

The long-term trajectory of kidney function recovery or decline for survivors of critical illness is incompletely understood. Characterising changes in kidney function after critical illness and associated episodes of acute kidney injury (AKI), could inform strategies to monitor and treat new or progressive chronic kidney disease. We assessed changes in estimated glomerular filtration rate (eGFR) and impact of AKI for 1301 critical care survivors with 5291 eGFR measurements (median 3 [IQR 2, 5] per patient) between hospital discharge (2004–2008) and end of 7 years of follow-up. Linear mixed effects models showed initial decline in eGFR over the first 6 months was greatest in patients without AKI (− 9.5%, 95% CI − 11.5% to − 7.4%) and with mild AKI (− 12.3%, CI − 15.1% to − 9.4%) and least in patients with moderate-severe AKI (− 4.3%, CI − 7.0% to − 1.4%). However, compared to patients without AKI, hospital discharge eGFR was lowest for the moderate-severe AKI group (median 61 [37, 96] vs 101 [78, 120] ml/min/1.73m^2^) and two thirds (66.5%, CI 59.8–72.6% vs 9.2%, CI 6.8–12.4%) had an eGFR of < 60 ml/min/1.73m^2^ through to 7 years after discharge. Kidney function trajectory after critical care discharge follows a distinctive pattern of initial drop then sustained decline. Regardless of AKI severity, this evidence suggests follow-up should incorporate monitoring of eGFR in the early months after hospital discharge.

## Introduction

Globally, hospital and community-based health services treat an increasing number of patients who survived an episode of critical illness^1,2^. After an intensive care unit (ICU) admission, patients have increased morbidity and mortality^2,3^, thought to be caused by the progression of chronic diseases^4,5^ and the long-term effects of acute illnesses such as sepsis^6,7^, and acute kidney injury (AKI)^8,9^. As post ICU follow-up becomes embedded within the healthcare services^10^, there are increasing opportunities to intervene to improve outcomes after critical illness^11,12^. However, the remit of follow-up services remains unclear and more research is required to help develop the appropriate strategies to address the health of patients after critical illness^11,13^.

Monitoring of kidney function after episodes of AKI has been suggested as a potential area for intervention^14^. Over half of the patients who are admitted to ICU experience an episode of AKI and epidemiological research has shown a consistent association with chronic kidney disease (CKD), end stage kidney disease, and death. Despite the potential benefit, patients who experience the most severe form of AKI requiring acute dialysis rarely receive specialist follow-up^15,16^. A lack of consensus on best practice after AKI episodes reflects the limitations of the current epidemiological research. Previous studies have focussed on outcomes such as requirement for long-term dialysis or a percentage decline in kidney function^17,18^, consequently ignoring trajectories provided by repeated biochemical measurements. Stratifying patients for follow-up using prediction models of advanced CKD after AKI are attractive^19^ but remain unproven clinically, and importantly, as the risk of death is higher than the need for dialysis^20^, may not capture patients who would benefit from proven interventions earlier. A reliable understanding of kidney function trajectory after critical illness modelled on repeated estimated glomerular filtration rate (eGFR) results^21^, as suggested for the monitoring of CKD progression^22,23^, is needed to identify high risk patients early and allow health services to best allocate precious resources.

To inform the development of strategies to monitor and treat new or progressive CKD after ICU discharge, the aims of this project were: (i) to model the change in kidney function for patients discharged alive from hospital after critical illness; (ii) to compare patients diagnosed with and without AKI during critical illness and to investigate the impact of AKI on long term kidney function up to 7 years after hospital discharge; and (iii) to explore whether features of critical illness such as the severity of AKI or extent of early recovery of kidney function affected the subsequent trajectory of eGFR.

## Results

Between 2004 and 2008, 4445 patients were admitted to ICU. Their primary reasons for admission were sepsis (29.1%), respiratory disease (28.2%), cardiovascular disease (14.4%), post-surgery (11.2%), AKI (5.3%), obstetric related disease (3.2%) and other causes (8.6%). Their mean length of stay in ICU was 8 days (SD 7.6).

Of 3090 patients discharged alive, we excluded 156 patients with pre-existing end stage kidney disease. Of the remaining 2934 patients, 1416 (48%) had no AKI, 729 (25%) mild AKI and 789 (27%) moderate to severe AKI whilst in ICU. Patients with moderate to severe AKI had a median age of 64 [IQR 51, 74] years, 37% were female and the median baseline eGFR was 72 ml/min/1.73 m^2^ [45, 75]. Patients without a diagnosis of AKI were younger, had a higher baseline eGFR and a higher proportion were female (Table 1).

**Table 1:** Patient characteristics by maximum acute kidney injury stage during intensive care unit admission.

	All	No AKI	Mild AKI	Moderate to severe AKI
**All patients (n = 2934)**				
All	2934	1416 (48%)	729 (25%)	789 (27%)
Female gender	1108 (38%)	584 (41%)	235 (32%)	289 (37%)
Age	60 [43, 72]	54 [38, 68]	64 [50, 74]	64 [51, 74]
Pre-ICU creatinine (n = 2178)	84 [67, 113]	76 [61, 92]	95 [76, 131]	99 [77, 144]
Pre-ICU eGFR	75 [60, 86]	75 [74, 96]	75 [51, 75]	72 [45, 75]
Pre-existing health conditions^a^	310 (11%)	132 (9%)	71 (10%)	107 (14%)
APACHE II score (n = 2847)	15 [11, 19]	13 [9, 16]	16 [12, 19]	19 [15, 23]
SOFA score (n = 2902)	5 [2, 7]	3 [2, 5]	5 [3, 7]	7 [5, 9]
Mechanical ventilation	2261 (77%)	1073 (76%)	618 (85%)	570 (72%)
Maximum number of organ failures (n = 2911)	3 [2, 3]	2 [2, 3]	3 [2, 3]	3 [2, 4]
**Patients with eGFR available during follow up (n = 1301)**				
All	1301	642 (49%)	303 (23%)	356 (27%)
Female gender	528 (41%)	288 (45%)	106 (35%)	134 (38%)
Age	58 [43, 70]	54 [40, 68]	61 [48, 72]	61 [47, 70]
Pre-ICU creatinine [μmol/L] (n = 1123)	80 [65, 105]	74 [61, 89]	94 [74, 124]	87 [68, 132]
Pre-ICU eGFR	75 [60, 94]	79 [70, 102]	73[50, 82]	73[51, 87]
Pre-existing health conditions^a^	166 (13%)	73 (11%)	39 (13%)	54 (15%)
APACHE II score (n = 1264)	15 [11, 19]	13 [10, 16]	16 [12, 19]	18 [15, 22]
SOFA score (n = 1286)	4 [2, 7]	3 [1, 5]	5 [3, 7]	7 [5, 10]
Mechanical ventilation	968 (74%)	458 (71%)	248 (82%)	262 (74%)
Maximum organ failure (n = 1289)	3 [2, 3]	2 [2, 3]	3 [2, 3]	3 [2, 4]

All values recorded at intensive care unit admission unless specified.

*AKI* acute kidney injury, *ICU* intensive care unit, *APACHE II* Acute Physiology And Chronic Health Evaluation II, *SOFA* sequential organ failure assessment, *eGFR* estimated glomerular filtration rate.

^a^Recorded according to the APACHE score chronic organ insufficiency.

Overall 1301 patients had 5291 eGFR measurements (median 3 [2, 5] per patient) routinely available between hospital discharge and 7 years of follow. Of these, 642 (49%) had had no diagnosis of AKI, 303 (23%) had mild AKI and 356 (27%) had moderate to severe AKI whilst in ICU (Table 1). Patients with no eGFR results available during follow-up were older, had a higher pre-ICU creatinine, lower pre-ICU eGFR, were less likely to have pre-existing conditions but more likely to have required mechanical ventilation (e-Table 1).

### Longitudinal modelling

**Figure 1:**
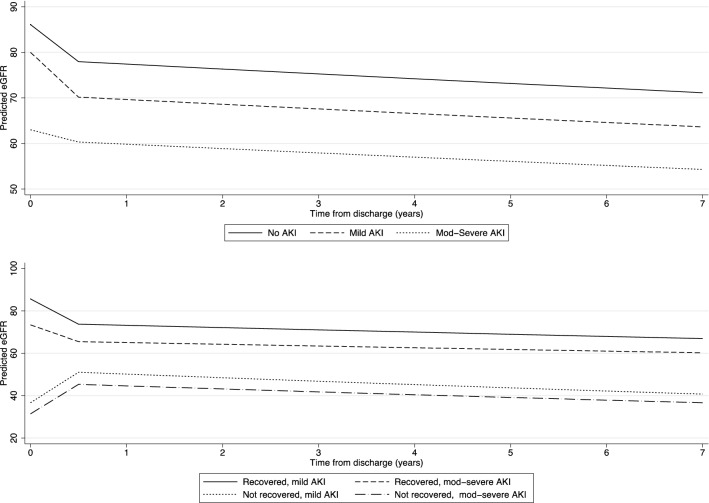
Predicted mean estimated glomerular filtration rate by acute kidney injury level and recovery status. Predicted eGFR was estimated using linear mixed effects models for log (eGFR) with a split slope for time, allowing the rate of change to differ in the first 6 months after discharge as compared to longer term. Lines shown represent fixed effects for a 55 years old male with a baseline eGFR of 75 and no pre-existing conditions and are shown on the original eGFR scale.

Overall, eGFR results at each time point from discharge to 7 years showed an initial rapid decline followed by slower decline over time (e-Fig. 2 and e-Fig. 3). Discharge eGFR was lowest for the moderate to severe AKI group (61 [37, 96] ml/min/1.73m^2^) followed by mild AKI (78 [56, 107] ml/min/1.73m^2^) and highest in patients with no AKI diagnosis (101 [78, 120] ml/min/1.73m^2^) during ICU admission. The results of the model for log eGFR by AKI level are summarized in Table 2 and Fig. 1. The initial decline in eGFR in the first 6 months after hospital discharge was highest for the no AKI [− 9.5% from 0 to 6 months, 95% confidence interval (CI) − 11.5% to − 7.4%] and mild AKI cohort (− 12.3%, 95% CI − 15.1% to − 9.4%). After 6 months, all groups declined at a similar rate of around − 1.5% per year (*p* = 0.955 for interaction).

**Table 2:** Multivariable model for association between maximum acute kidney injury stage and post-discharge eGFR results.

	% change in eGFR (95% CI)	*p* value
**All patients (n = 1301)**		
Age (per 1 year increase)	− 0.7 (− 0.8, − 0.5)	< 0.001
Female gender	0.1 (− 3.5, 3.9)	0.939
Baseline eGFR (per 10 increase)	10.5 (9.5, 11.5)	< 0.001
Pre− existing health conditions^a^	− 3.7 (− 8.8, 1.7)	0.173
**Change from 0 to 6 months**		
No AKI	− 9.5 (− 11.5, − 7.4)	< 0.001
Mild AKI	− 12.3 (− 15.1, − 9.4)	
Moderate− severe AKI	− 4.3 (− 7.0,− 1.4)	
**Annual rate of change 6 months–7 years**		
No AKI	− 1.4 (− 2.2, − 0.6)	0.955
Mild AKI	− 1.5 (− 2.6, − 0.3)	
Moderate− severe AKI	− 1.6 (− 2.7, − 0.5)	

Log (eGFR) was modelled using linear mixed effects models with a split slope for time, allowing the rate of change to differ in the first 6 months after discharge as compared to longer term. Exponentials of model co-efficient were calculated to provide estimates of the effect of the dependent variables on the % change in eGFR. There was no evidence in of a difference in the effect of recovery by AKI level up to 6 months or from 6 months to 7 years (p for interaction = 0.256 and 0.218 , respectively).

*AKI* acute kidney injury ICU, *eGFR* estimated glomerular filtration rate.

^a^Recorded according to the APACHE score chronic organ insufficiency.

After 7 years of follow-up, 833 patients were still alive. Among these patients the modelled eGFR was < 60 ml/min/1.73m^2^ in 34.6% (95% CI 28.1–41.6%) of patients with mild AKI, in 66.5% (95% CI 59.8–72.6%) of patients with moderate to severe AKI and in 9.2% (95% CI 6.8% to 12.4%) of those with no AKI. In sensitivity analyses, modelling was repeated using only follow-up data from patients alive at 7 years after discharge; trends were similar to the main analysis (e-Fig. 4 and e-Table 4).

We repeated longitudinal modelling to assess the effect of the exposure of non-recovery of AKI on changes in eGFR over time. Of the 659 patients with AKI, 65 (10%) did not recover kidney function by hospital discharge. Patients without kidney recovery were older (median 66 [52, 73] vs 61 [47, 71] years) and a higher proportion had moderate to severe AKI compared to mild AKI (80% vs 51%) (e-Table 2). For the non-recovery group, eGFR improved over the first 6 months but then fell at a greater rate than in the group who recovered kidney function (*p* = 0.025 for interaction). For the non-recovery group, the average long-term decline was − 3.4% (CI − 6.3% to − 0.4%) per year for those with mild AKI and − 3.2% (CI − 5.8%, − 0.5%) for moderate to severe AKI compared to − 1.5% (− 2.6% to − 0.3%) and − 1.3% (− 2.4% to − 0.1%), respectively, for the recovered group (etable 3 and Fig. 1).

### Survival analysis

Overall 1199 deaths were recorded during 7 years of follow up with the cumulative risk of death of 10.6% (95% CI 9.6–11.8%) at 6 months, 35.0% (95% CI 33.3–36.7%) at 5 years and 40.9% (95% CI 39.1–42.7%) at 7 years. Forty-six patients started chronic dialysis during follow-up and 1227 started chronic dialysis and/or died giving a cumulative risk of death or dialysis of 11.7% (95% CI 10.6–12.9%) at 6 months, 36.0% (34.3–37.7%) at 5 years and 41.8% (95% CI 40.1–43.6%) at 7 years.

**Figure 2:**
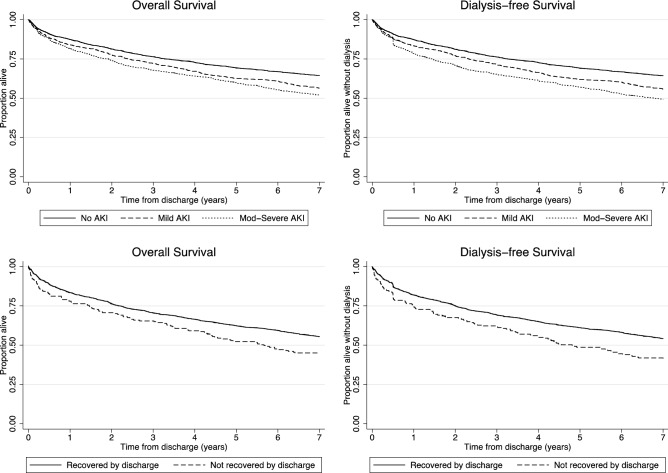
Cumulative survival and dialysis free survival by acute kidney injury level and recovery status at discharge. Log rank test for unadjusted differences in overall survival by AKI level: *p* =  < 0.001, by recovery group: *p* = 0.004, in dialysis free survival by AKI: *p* =  < 0.001 and by recovery group: *p* =  < 0.001.

In univariable analyses there were differences in survival and dialysis-free survival by AKI stage and by kidney function recovery status in patients with AKI (Fig. 2). In multivariable analysis, the moderate to severe AKI cohort had a 19% increase in the hazard of death or dialysis (HR 1.19, 95% CI 1.04–1.36) compared with no AKI (Table 3). There was no increase in the hazard of death or dialysis for patients with mild AKI. Among patients with AKI, non-recovery of kidney function by discharge was associated with an increase in the hazard of death or chronic dialysis (HR 1.30, 95% CI 1.06–1.60) (e-table 5).

**Table 3:** Multivariable analysis for association between acute kidney injury and overall survival and dialysis free survival.

	Hazard of death		Hazard of death or dialysis	
	HR (95% CI)	*p* value	HR (95% CI)	*p* value
**All patients (n = 2934)**				
Age	1.04 (1.04, 1.05)	< 0.001	1.04 (1.04, 1.05)	< 0.001
Female gender	0.94 (0.84, 1.06)	0.339	0.94 (0.84, 1.06)	0.341
Pre-existing health conditions^a^	2.17 (1.84, 2.56)	< 0.001	2.20 (1.87, 2.59)	< 0.001
**Max AKI level**				
None	1	0.166	1	0.015
Mild	0.98 (0.85, 1.13)		0.99 (0.86, 1.14)	
Moderate—severe	1.12 (0.97, 1.28)		1.19 (1.04, 1.36)	

All models were additionally adjusted for baseline eGFR which was significant in all models (*p* < 0.001). eGFR was modelled using second degree fractional polynomials (FP(3,3) provided best fit in all models) to allow for the non-linear relationship with hazard of death. The nature of his relationship is illustrated in Figure S6; hazard of death was highest among patients with low or high eGFR, and lowest among patients with eGFR between 70 and 100.

*AKI* acute kidney injury ICU, *HR* hazard ratio, *CI* confidence interval.

^a^Recorded according to the APACHE score chronic organ insufficiency.

For the 1301 patients with eGFR data during follow-up who were included in the longitudinal modelling, the cumulative risk of death was 36.0% (95% CI 33.4–38.7%) at 7 years and death or dialysis was 36.1% (95% CI 33.9–39.1%). In this group, the evidence suggested moderate-severe AKI was possibly associated with an increased hazard of death or chronic dialysis (HR 1.23, 95% CI 0.99–1.52) (e-table 6).

In an exploratory joint analysis of longitudinal eGFR data and dialysis free survival (death or chronic dialysis) data, a 1% decline in eGFR after hospital admission was potentially associated with 0.3% (95% CI 0.01–0.5%) increase in risk of death or need for dialysis after adjustment (age, gender, pre-existing health conditions and baseline eGFR) (e-Table 7).

## Discussion

Our long-term analysis of patients discharged alive from hospital after an ICU admission showed that kidney function fell substantially in the following 7 years. In the group diagnosed with no or mild AKI during ICU admission, eGFR decreased by around 10% in the first 6 months followed by a shallower but sustained decline. In comparison, patients with moderate to severe AKI were more likely to have a lower eGFR prior to hospital admission as well as at hospital discharge and throughout the seven year follow up period. Over a third of patients died in the 5 years after hospital discharge and the data suggested several associations with trajectory of kidney function. Firstly, the decline in eGFR after hospital discharge was potentially associated with late mortality or requirement for chronic dialysis in the exploratory joint model. Secondly, the evidence suggests patients with the lowest baseline and follow-up kidney function, characteristic of the moderate to severe AKI group, had an estimated 20% increase in the hazard of death or need for dialysis over 7 years. Lastly, patients who did not recover kidney function had the greatest decline in eGFR over follow-up and the worst long-term dialysis-free survival.

Follow-up decisions regarding long-term monitoring of kidney function should be informed by eGFR measurements taken after hospital discharge. A post-ICU follow-up clinic, reviewing patients at 3 months after ICU discharge^10^, is an appropriate juncture for clinicians to assess long term kidney function, plan appropriate hospital or community follow-up, and control cardiovascular risk factors. Management of the decline of eGFR and the associated risk factors is a potential area for intervention to address the considerable excess long-term morbidity and mortality experienced by patients discharged from hospital after critical illness. Our data show a substantial decline in kidney function in the first 6 months after hospital discharge which suggests that creatinine results at hospital discharge can be misleading. This cohort, on average had an eGFR > 60 ml/min/1.73m^2^ based on serum creatinine results and were therefore discharged to the community with assumed near-normal kidney function.

Our findings are consistent with previous studies that showed deterioration of kidney function in patients who had survived an ICU admission and worse outcomes after more severe AKI^8^. However, in the context of kidney function after AKI, decline is often defined using cut-off values such as percentage decrease in kidney function^8^ or by identification of a CKD related code after hospital discharge^24^. These studies highlight the potential risk of kidney function decline yet make planning an intervention strategy difficult which may explain the low level of routine follow-up after AKI^25^. Modelling of repeated measures data has been embraced by investigators undertaking longitudinal studies in CKD^23^. There is the potential that similar strategies could improve clinical management after AKI. Additionally, patients who do not recover kidney function following an AKI episode have repeatedly been shown to have an increased risk of subsequent CKD and worse survival^26–28^.

The current best practice guidance for follow-up after hospital associated AKI^29^ suggests planning a review on hospital discharge based on eGFR. Our study suggests that this is inappropriate for ICU survivors, may be misleading and may present a missed opportunity to intervene. The use of repeated measures data should inform future strategies and research into follow-up monitoring of kidney function, itself a research priority for health services^30^.

There are several mechanistic pathways that can help explain the results of this study. The observed rapid decline in eGFR during the first 6 months after hospital discharge is likely attributed to the confounding effect of acute skeletal muscle wasting on serum creatinine and the overestimation of kidney function^31,32^. Only when muscle volume has recovered in the months after acute illness can more reliable estimates of kidney function be made using creatinine-based formula.

Recent studies have highlighted the effect of the maladaptive repair process after AKI on subsequent progression of kidney function decline^33^. Research in mice has shown that tubulointerstitial fibrosis, a core pathway in maladaptive repair, is prevalent after AKI and may take time to manifest resulting in apparent initial recovery^34,35^. An increase in urinary concentration of dickkopf-3 (DKK3), a urinary cytokine and modulator of the tubulointerstitial fibrotic process, has been linked to a fall in GFR after hospital discharge post cardiac surgery, including for patients with apparently normal GFR^36^. The subsequent plateau of eGFR decline which we observed may represent the new baseline kidney function. As elucidated in animal models, a further decline in GFR then requires a subsequent “second hit” to cause further kidney damage^37^. For those patients with more severe AKI, the process of maladaptive repair may be more profound^33^, thus explaining the less dramatic decline but lower average eGFR overall observed in our cohort.

The strong epidemiological association between increasing cardiovascular events and fall in GFR, widely reported in patients with CKD^20,38^, has several credible biological pathways which may be present in ICU survivors, too. Potential mechanisms of AKI induced cardiac dysfunction have been found in models of AKI and include endothelial dysfunction^39^, microcirculatory abnormalities^40^ and human artery calcification^41,42^. In addition, reconciliation of cardioprotective drugs, including angiotensin converting enzyme inhibitors post AKI is often perceived as challenging and may contribute to cardiovascular complications^43^. Management of cardiovascular risk could be a potential target to reduce the excess mortality and morbidity of ICU survivors.

Our study has several limitations. First, eGFR results were only available for patients followed up our institution, albeit a large tertiary NHS referral centre for south east London. Reassuringly, the incidence of AKI and long-term mortality are similar to national registry-based studies^1,2^. Second, these findings are at risk of ascertainment bias as patients attending outpatient clinics for a blood test would do so for a reason. Although these results represent real world data, they are vulnerable to confounding. To help reduce this risk, the random effects modelling framework was chosen to account for both informative missing measurements and different numbers of observations at different time points^23^. Furthermore, we had complete data for starting chronic dialysis and mortality. Third, baseline creatinine results were not available for all patients and instead had to be calculated using the current recommended method^44^. To reduce the confounding effect of pre-existing CKD, we modelled on hospital discharge creatinine which we had complete data for and included baseline eGFR as a covariate. Fourth, we did not have complete data on urine protein excretion, another important marker of kidney disease. Again, this reflects the real world setting. Fifth, due to low numbers of patients, our renal recovery analysis is hypothesis generating and we were unable to look at incidence of acute kidney disease (AKD), an important interim diagnosis, with increasingly recognised implications for long-term kidney function^45^. However, we note that the specific criteria for AKD have not been agreed^13^. Finally, we were unable to collect data on specific cardiovascular events or the cause of death, limiting the interpretation of the impact of kidney function decline on outcomes.

Our study has several strengths. We used a prespecified analysis plan and a robust methodology to harness repeated measures data of eGFR trajectory in ICU survivors and provide novel data to inform potential follow-up strategies. The mixed effects modelling framework accounts for eGFR measurement error where the true eGFR can be 30% above and below this for up to a quarter of patients^20^. The use of the joint model in the exploratory analysis strengthened the findings of survival analysis, adding important evidence linking falls in eGFR with worse outcomes after ICU admission.

## Conclusions

In a large cohort of ICU survivors, we have shown a substantial decline in kidney function in the first 6 months after hospital discharge. This evidence suggests follow-up after AKI needs to incorporate regular monitoring of kidney function in the months after hospital discharge as eGFR measurements within this timeframe are more informative for clinicians of eGFR decline. In addition, the months after hospital discharge are when newer interventions to monitor or prevent a decline in kidney function should be targeted to address the associated increased risk of poor outcomes.

## Methods

### Study design and participants

We did a retrospective observational cohort study including 3090 adult patients (> 18 years) who were admitted to a 43-bed multi-disciplinary level 3 ICU in a university hospital in the UK between 2004 and 2008 and left hospital alive. We excluded patients with end stage kidney disease or a kidney transplant prior to admission to ICU. We followed the STROBE guidelines for reporting of cohort studies (see supplement).

### Data source

We linked ICU electronic medical records with routinely available data on the institutional electronic patient record system. Date of death was obtained from the medical health records and the UK National Health Service registry. Start date of chronic dialysis or renal transplant was obtained from the UK Renal Registry. The project had institutional approval and was registered as service evaluation (GSTT Project number 3337). We had waiver of consent for use of routinely available data from Guy’s and St Thomas’s audit and service evaluation team.

### Exposures and outcome

The exposure was AKI during ICU admission. AKI was staged according to the serum creatinine criteria of the Kidney Disease Improving Global Outcome (KDIGO) classification^44^. Hereafter, we refer to KDIGO AKI Stage 1 as mild AKI and KDIGO AKI Stage 2 and 3 as moderate to severe AKI. When available, we recorded a baseline creatinine result within 7–365 days before ICU admission^28^. When the pre-ICU admission value was unavailable, we assumed near normal kidney function as per KDIGO guidance^44^, i.e. a baseline eGFR of 75 ml/min/1.73 m^2^. We used hospital discharge creatinine values as the first creatinine to be included in longitudinal modelling.

We recorded baseline demographics for all patients including, age, gender, Acute Physiology Chronic Health Evaluation II (APACHE II) and Sequential Organ Failure Assessment (SOFA) scores on admission to ICU. We recorded the APACHE II advanced comorbid diseases including presence of advanced liver disease, chronic respiratory disease with severe limitation of exercise tolerance, New York Heart Association class IV heart failure or need for immunosuppression.

The primary outcome was kidney function determined as eGFR during follow up. Measurements were recorded from the hospital electronic patient record system at 6 months through to 7 years after hospital discharge. Serum creatinine results at hospital discharge were collected and compared with baseline values to establish whether patients had exhibited kidney function recovery. To determine whether early recovery of kidney function influenced long-term outcomes of AKI, renal recovery was defined as return of serum creatinine at discharge to pre-admission or computed baseline value. We recorded development of end stage kidney disease and patient survival up to 7 years following discharge from hospital until June 2016.

### Statistical analysis

Patients were categorised into three groups: no AKI, mild AKI and moderate to severe AKI. A mixed effects model was used to explore the relationship between AKI and log eGFR up to 7 years after discharge (supplementary methods). To assess the effect of recovery of kidney function, we compared AKI patients who had recovered (ie return of serum creatinine to baseline value) and not recovered kidney function by hospital discharge and applied a similar model in patients with AKI only.

Survival time was calculated as time to death with observations censored at 7 years after discharge. Dialysis free survival time was calculated as time to death or chronic dialysis. Kaplan Meier methods were applied to estimate overall, and dialysis free survival and log-rank and Cox proportional hazards models were used to compare survival across AKI groups and in those who had and had not recovered kidney function by hospital discharge. Adjustment for confounding variables was based on biological plausibility and previous studies^8^. Finally, a joint model was used to combine the linear mixed model and Cox survival model to explore the association between longitudinal changes in eGFR during follow up and risk of death or chronic dialysis^46^. In sensitivity analyses, the linear mixed models were fitted to the subset of patients alive at 7 years. Analysis was carried out using Stata 15IC and R version 3.6.1 using the JM package.

## Supplementary Information

Supplementary Information.

## Data Availability

Code can be obtained from the corresponding author on request. No additional data available.
