# Supplementary material for: Long-term kidney function of patients discharged from hospital after an intensive care admission: observational cohort study
Source: Sci Rep. 2021 May 11;11:9928. doi: 10.1038/s41598-021-89454-3 (PMC8113423; doi:10.1038/s41598-021-89454-3)
Supplement: Supplementary file 1 — Supplementary Information. [file 41598_2021_89454_MOESM1_ESM.docx]

**Supplementary material:**

**Long-term kidney function of patients discharged from hospital after an intensive care admission: observational cohort study**

Ryan W Haines (0000-0002-4864-2825), Jonah Powell-Tuck, Hugh Leonard, Siobhan Crichton, Marlies Ostermann

Supplementary methods

The model included patient specific random intercepts and slopes and, assumed a first-order autoregressive process residual correlation structure to control for repeated measurements. Linearity of the relationship between time and log eGFR was explored and suggested a difference in rate of change in the first 6 months as compared to longer term and therefore a split slope regression model was used with fixed effects taking the following structure:

*formula <- log eGFR ~ time + time(6m-7y) + AKI category + AKI category#time + AKI category#time(6m-7y) + baseline age + gender + major comorbidity + baseline eGFR*

Supplementary tables and figures

eTable 1: Patient characteristics of those with and without kidney function follow-up data.

|  | All | No eGFR during follow up | ≥ 1 eGFR during follow up |
| --- | --- | --- | --- |
| All | 2934 | 1633 | 1301 |
| Male gender | 1825 (62%) | 1052 (64%) | 773 (59%) |
| Age | 60 [43,72] | 62 [44,74] | 58 [43,70] |
| Pre-ICU creatinine (n=2178) | 84 [67,113] | 89 [70,122] | 80 [65,105] |
| Pre-ICU eGFR (n=2934) | 75 [60,86] | 75 [59,78] | 75 [60,94] |
| Preexisting health conditions* | 310 (11%) | 144 (9%) | 166 (13%) |
| APACHE score (n=2847) | 15 [11,19] | 15 [11.19] | 15 [11,19] |
| SOFA score (n=2902) | 5 [2,7] | 5 [3,7] | 4 [2,7] |
| Mechanical ventilation | 2261 (77%) | 1293 (79%) | 968(74%) |
| Maximum number of organ failures (n=2911) | 3 [2,3] | 3 [2,3] | 3 [2,3] |

All values recorded at intensive care unit admission unless specified.

*Recorded according to the APACHE score chronic organ insufficiency.

Abbreviations: ICU, intensive care unit; APACHE II, Acute Physiology And Chronic Health Evaluation II; SOFA, sequential organ failure assessment; eGFR, estimate glomerular filtration rate

eTable 2: Characteristics of patients with acute kidney injury who did and did not have recovery of kidney function by hospital discharge

|  | All patients | | ≥ 1 eGFR during follow up | |
| --- | --- | --- | --- | --- |
|  | Recovered | Not recovered | Recovered | Not recovered |
| All | 1318 | 191 | 594 | 65 |
| Female gender | 447 (34%) | 75 (39%) | 215 (36%) | 25 (38%) |
| Age | 64 [50,74] | 68 [57,75] | 61 [47,71] | 66 [52,73] |
| Pre-ICU creatinine | 98 [75,138] | 99 [75,147] | 90 [70,124] | 90[67,133] |
| Pre-ICU eGFR | 75 [64,75] | 75 [69,75] | 75[66,76] | 75[61,75] |
| Preexisting health conditions* | 151 (12%) | 24 (13%) | 83 (14%) | 10 (15%) |
| APACHE score | 17 [13,21] | 19 [15,23] | 17 [13,21] | 17 [14,23] |
| SOFA score | 6 [4,8] | 6[5,9] | 6 [4,8] | 6[4,9] |
| Mechanical ventilation | 1058 (80%) | 126 (66%) | 467 (79%) | 43 (66%) |
| Maximum number of organ failures | 3 [2,4] | 3 [2,3] | 3 [2,4] | 3 [2,4] |
| Moderate-severe AKI | 630 (48%) | 150 (79%) | 304 (51%) | 52 (80%) |

Recovery status at discharge unavailable for 9 patients with AKI

All values recorded at intensive care unit admission unless specified.

*Recorded according to the APACHE score chronic organ insufficiency.

Abbreviations: ICU, intensive care unit; APACHE II, Acute Physiology And Chronic Health Evaluation II; SOFA, sequential organ failure assessment; eGFR, estimate glomerular filtration rate; AKI, acute kidney injury

eTable 3: Multivariable model for association between acute kidney injury recovery and post-discharge eGFR results.

|  | **% change in eGFR (95% CI)** | **p-value** |
| --- | --- | --- |
| **Patients with AKI (n=659)** | | |
| Age | -0.6 (-0.8,-0.5) | <0.001 |
| Female gender | 4.0 (-1.5, 9.6) | 0.159 |
| Baseline eGFR | 1.3 (1.1, 1.4) | <0.001 |
| Pre-existing health conditions | -5.4 (-12.4, 2.2) | 0.156 |
| Change from 0-6 months |  |  |
| Recovered by discharge | -23.9 (-16.9, -10.9) | <0.001* |
| Recovered by discharge, moderate-severe AKI | -40.9 (-13.7, -7.8) |  |
| Not recovered by discharge | 39.2 (28.0, 51.3) |  |
| Not recovered by discharge, moderate-severe AKI | 44.3 (33.9, 55.4) |  |
| Annual rate of change 6 months – 7 years |  |  |
| Recovered by discharge, mild AKI | -1.5 (-2.6, -0.3) | 0.025* |
| Recovered by discharge, moderate-severe AKI | -1.3 (-2.4, -0.1) |  |
| Not recovered by discharge, mild AKI | -3.4 (-6.3, -0.4) |  |
| Not recovered by discharge, moderate-severe AKI | -3.2 (-5.8, -0.5) |  |

There was no evidence in of a difference in the effect of recovery by AKI level up to 6 months or from 6 months to 7 years (p for interaction =0.256 and 0.218, respectively)

Abbreviations: AKI, acute kidney injury; eGFR, estimated glomerular filtration rate; CI, confidence interval

eTable 4: Multivariable model for association between acute kidney injury level post-discharge eGFR measurements in patients alive at 7 years.

|  | **% change in eGFR (95% CI)** | **p-value** |
| --- | --- | --- |
| **All patients (n=833)** | | |
| Age (per 1 year increase) | -0.7 (-0.8, -0.5) | <0.001 |
| Female gender | -2.0 (-6.0, 2.1) | 0.327 |
| Baseline eGFR (per 10 increase) | 8.5 (7.2, 9.6) | <0.001 |
| Pre-existing health conditions | -5.0 (-10.9, 1.3) | 0.119 |
| Change from 0 - 6 months |  |  |
| No AKI | -8.1 (-10.4, -5.8) | <0.001 |
| Mild AKI | -11.3 (-14.5, -7.9) |  |
| Moderate-severe AKI | -0.3 (-3.6, 3.3) |  |
| Annual rate of change 6 months – 7 years |  |  |
| No AKI | -1.4 (-2.1, -0.6) | 0.818 |
| Mild AKI | -0.9 (-2.1, 0.2) |  |
| Moderate-severe AKI | -1.3 (-2.3, -0.2) |  |
| **Patients with AKI (n=659)** | | |
| Age | -0.6 (-0.8,-0.5) | <0.001 |
| Female sex | 4.0 (-1.5, 9.6) | 0.159 |
| Baseline eGFR | 1.3 (1.1, 1.4) | <0.001 |
| Pre-existing health conditions | -5.4 (-12.4, 2.2) | 0.156 |
| Change from 0-6 months |  |  |
| Recovered by discharge | -12.4 (-15.8, -8.9) | <0.001* |
| Recovered by discharge, moderate-severe AKI | -8.4 (-11.9, -4.7) |  |
| Not recovered by discharge | 61.7 (61.7, 79.7) |  |
| Not recovered by discharge, moderate-severe AKI | 69.2 (54.0, 85.8) |  |
| Annual rate of change 6 months – 7 years |  |  |
| Recovered by discharge, mild AKI | -0.9 (-2.1, 0.2) | 0.039* |
| Recovered by discharge, moderate-severe AKI | -0.9 (-2.0, 0.3) |  |
| Not recovered by discharge, mild AKI | -3.9 (-6.8, -0.9) |  |
| Not recovered by discharge, moderate-severe AKI | -3.8 (-6.3, -1.2) |  |

Log (eGFR) was modelled using linear mixed effects models with a split slope for time, allowing the rate of change to differ in the first 6 months after discharge as compared to longer term. Exponentials of model co-efficient were calculated to provide estimates of the effect of the dependent variables on the % change in eGFR. * p-value for difference in slopes by recovery status, after adjustment for AKI level. There was no evidence in of a difference in the effect of recovery by AKI level up to 6m or from 6m to 7 y (p for interaction =0.134 and 0.571, respectively

Abbreviations: AKI, acute kidney injury; eGFR, estimated glomerular filtration rate; CI, confidence interval

eTable 5: Multivariable analysis for association between acute kidney injury and overall survival and dialysis free survival in the subset of patients with AKI by recovery status.

|  | | **Hazard of death** | | | | **Hazard of death or dialysis** | | |  |
| --- | --- | --- | --- | --- | --- | --- | --- | --- | --- |
|  | | **HR (95% CI)** | | **p-value** | | **HR (95% CI)** | | **p-value** |  |
| **Patients with AKI (n=1509)** | | | | | | | | | |
| Age | 1.04 (1.04,1.05) | | <0.001 | | 1.04 (1.03,1.04) | | <0.001 | | |
| Female gender | 1.01 (0.87,1.19) | | 0.855 | | 1.01 (0.87,1.18) | | 0.879 | | |
| Pre-existing health conditions^a^ | 2.05 (1.63,2.57) | | <0.001 | | 2.00 (1.61,2.51) | | <0.001 | | |
| Moderate-severe AKI | 1.10 (0.94,1.28) | | 0.237 | | 1.13 (0.97,1.64) | | 0.107 | | |
| Renal function not recovered by hospital discharge | 1.21 (0.98,1.50) | | 0.082 | | 1.30 (1.06, 1.60) | | 0.014 | | |

There was no evidence of any difference in the association between non-recovery and hazard of death or hazard of dialysis or death by max AKI level (p for interaction 0.264 and 0.419, respectively)

Abbreviations: AKI, acute kidney injury; eGFR, estimated glomerular filtration rate; CI, confidence interval

eTable 6: Multivariable analysis for association between acute kidney injury and overall survival and dialysis free survival in the subset of patient with eGFR data available during follow-up

|  | **Hazard of death** | | **Hazard of death or dialysis** | |
| --- | --- | --- | --- | --- |
|  | **HR (95% CI)** | **p-value** | **HR (95% CI)** | **p-value** |
| **All patients (n=1301)** | | | | |
| Age | 1.04 (1.04, 1.05) | <0.001 | 1.04 (1.04, 1.05) | <0.001 |
| Female | 0.93 (0.77, 1.12) | 0.460 | 0.91 (0.75, 1.09) | 0.304 |
| Pre-existing health conditions* | 1.85 (1.44, 2.38) | <0.001 | 1.86 (1.45, 2.39) | <0.001 |
| Max AKI level |  |  |  |  |
| None | 1 | 0.092 | 1 | 0.072 |
| Mild | 0.95 (0.75, 1.20) |  | 0.95 (0.75, 1.20) |  |
| Moderate - severe | 1.18 (0.95, 1.46) |  | 1.23 (0.99, 1.52) |  |
| **Patients with AKI (n=659)** | | | | |
| Age | 1.04 (1.03,1.05) | <0.001 | 1.04 (1.03,1.04) | <0.001 |
| Female | 1.04 (0.81,1.34) | 0.756 | 0.99 (0.76,1.27) | 0.897 |
| Pre-existing health condition | 1.69 (1.18,2.40) | <0.001 | 1.69 (1.19,2.40) | <0.001 |
| Mod-severe AKI | 1.20 (0.93,1.55) | 0.157 | 1.23 (0.96,1.59) | 0.101 |
| Not recovered by discharge | 1.00 (0.68,1.47) | 0.996 | 1.11 (0.76, 1.61) | 0.579 |

All models were additionally adjusted for baseline eGFR. eGFR was modelled using second degree fractional polynomials (FP(3,3) provided best fit in all models) to allow for the non-linear relationship with hazard of death.

There was no evidence of any difference in the association between non-recovery and hazard of death or hazard of dialysis or death by max AKI level (p for interaction 0.410 and 0.775, respectively)

Abbreviations: AKI, acute kidney injury; eGFR, estimated glomerular filtration rate; CI, confidence interval

eTable 7: Results for the joint modelling analysis of eGFR and time to death or death and/or dialysis.

|  | Death only | | | Death or dialysis | | |
| --- | --- | --- | --- | --- | --- | --- |
|  | Value | Std.Err | p-value | Value | Std.Err | p-value |
|  | Longitudinal sub-model | | | | | |
| (Intercept) | 3.969 | 0.059 | <0.001 | 3.969 | 0.059 | <0.001 |
| Age | -0.006 | 0.001 | <0.001 | -0.006 | 0.001 | <0.001 |
| Female | 0.001 | 0.019 | 0.969 | 0.001 | 0.019 | 0.971 |
| Baseline eGFR (per 10 increase) | 0.100 | 0.005 | <0.001 | 0.100 | 0.005 | <0.001 |
| Pre-existing health condition | -0.045 | 0.027 | 0.098 | -0.045 | 0.027 | 0.097 |
| Time 0 to 6m (per 1y increase) | -0.182 | 0.024 | <0.001 | -0.182 | 0.024 | <0.001 |
| Interaction: Mild AKI X time 0-6m | -0.065 | 0.039 | 0.096 | -0.065 | 0.039 | 0.092 |
| Interaction: Mod-severe AKI X time 0-6m | 0.104 | 0.037 | 0.004 | 0.105 | 0.037 | 0.004 |
| Time 6 months - 7 years (per 1y increase) | -0.015 | 0.003 | <0.001 | -0.016 | 0.003 | <0.001 |
| Mild AKI | -0.105 | 0.025 | <0.001 | -0.105 | 0.025 | <0.001 |
| Moderate - severe AKI | -0.260 | 0.024 | <0.001 | -0.260 | 0.024 | <0.001 |
|  | Survival sub-model | | | | | |
|  | Value | Std.Err | p-value | Value | Std.Err | p-value |
| Age | 0.044 | 0.004 | <0.001 | 0.042 | 0.004 | <0.001 |
| Female | -0.060 | 0.096 | 0.528 | -0.101 | 0.096 | 0.292 |
| Pre-existing health condition | 0.627 | 0.128 | <0.001 | 0.632 | 0.127 | <0.001 |
| Baseline eGFR<50 | 0.180 | 0.165 | 0.276 | 0.115 | 0.163 | 0.480 |
| Baseline eGFR>100 | 0.519 | 0.142 | 0.000 | 0.541 | 0.141 | 0.000 |
| log(eGFR) | -0.107 | 0.125 | 0.392 | -0.254 | 0.123 | 0.038 |

The longitudinal component of the joint model included a random intercept and random slope. Fixed effects for time allowed the change in log(eGFR) to vary by AKI severity up to 6 months then a common rate of decline was assumed. Baseline eGFR was initially included in the survival sub model using a second-degree fractional polynomial, found to provide the best in the univariate survival models. In the joint model, this was no longer significant, while inclusion of a linear term indicated an elevated risk of death with increasing baseline eGFR. We therefore categorised baseline eGFR using cut-offs driven by the univariate survival models (supplementary figure S6) to allow for a potential independent increased risk of death in those with initial very low or high pre-existing eGFR. In the death only model a 1% decrease in eGFR during follow up was associated with a non-significant 0.1% (100*exp(0.107/100) -1) (95% CI -0.1 to 0.4%) increase in hazard of death and in the death or dialysis model with a 0.3% (0.01 to 0.5) increase.

Abbreviations: AKI, acute kidney injury; eGFR, estimated glomerular filtration rate; CI, confidence interval; Std.Err, standard error

eFigure 1: Study flow diagram

Abbreviations: AKI, acute kidney injury; eGFR, estimated glomerular filtration rate

eFigure 2: Distribution of eGFR measurements over time

Abbreviations: eGFR, estimated glomerular filtration rate

eFigure 3: Relationship between average eGFR and time. Fractional polynomials (FP) were used to explore the nature of the relationship between log eGFR and time. From discharge, there was evidence that a second-degree FP provided a better fit than a first degree FP (=0.003) or a linear model (p<0.001). The best fitting curve is represented by the figure above, which shows an initial sharp decline in the first 6 months. When measurements at discharge were excluded there was no evidence of better fit compared to a simpler linear model when using first degree (p=0.999) or second degree (p=0.324) FPs to represent time from 6 months post discharge onwards.

eFigure 4: Predicted mean eGFR by acute kidney injury level and recovery status in patients alive at 7 years.

Predicted eGFR for was estimated using linear mixed effects models for log (eGFR) with a split slope for time, allowing the rate of change to differ in the first 6 months after discharge as compared to longer term. Lines shown represent fixed effects for a 55 years old male with a baseline eGFR of 75 and no pre-existing conditions and are shown on the original eGFR scale.

Abbreviations: AKI, acute kidney injury; eGFR, estimated glomerular filtration rate

eFigure 5: Cumulative survival and dialysis free survival by AKI level and recovery status at discharge in the subset of patient with eGFR data available during follow-up

Log rank test for unadjusted differences in overall survival by AKI level: p=0.009, by recovery group: p= 0.178, in dialysis free survival by AKI: p=0.001 and by recovery group: p=0.044

Abbreviations: AKI, acute kidney injury

eFigure 6: Association between baseline eGFR and hazard of death (left) and death or dialysis (right) among all patients.


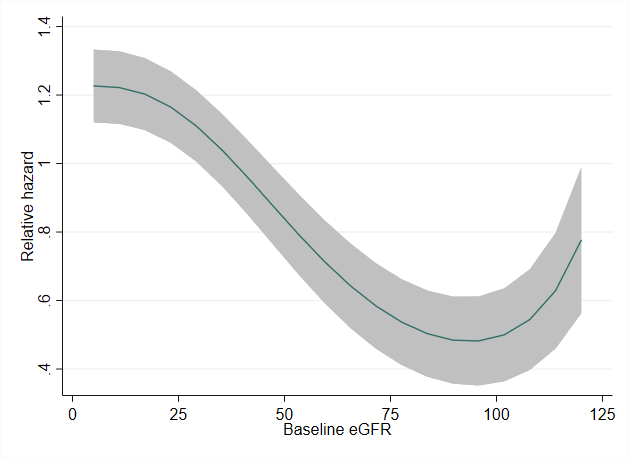

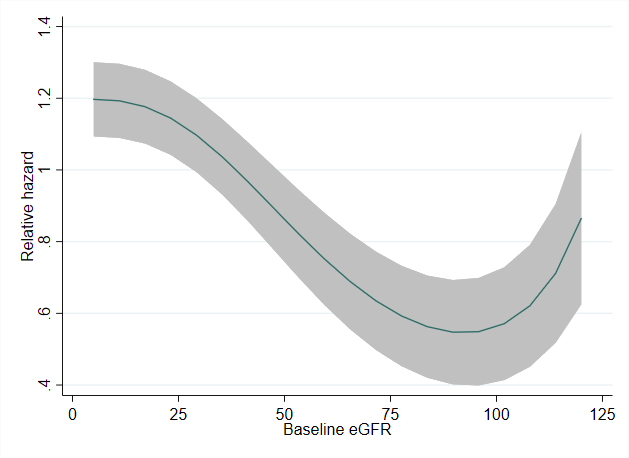


Abbreviations: eGFR, estimated glomerular filtration rate
